# Resistance to DDT in an Urban Setting: Common Mechanisms Implicated in Both M and S Forms of *Anopheles gambiae* in the City of Yaoundé Cameroon

**DOI:** 10.1371/journal.pone.0061408

**Published:** 2013-04-23

**Authors:** Billy Fossog Tene, Rodolphe Poupardin, Carlo Costantini, Parfait Awono-Ambene, Charles S. Wondji, Hilary Ranson, Christophe Antonio-Nkondjio

**Affiliations:** 1 Laboratoire de Recherche sur le Paludisme, Organisation de Coordination pour la lutte Contre les Endémies en Afrique Centrale, Yaoundé, Cameroon; 2 Faculty of Sciences, University of Yaoundé I, Cameroon; 3 Institut de Recherche pour le Développement, UMR IRD 224 Centre national de la recherche scientifique 5290 Université de Montpellier 1 Université de Montpellier 2, Maladies Infectieuses et Vecteurs Écologie, Génétique, Évolution et Contrôle, Montpellier, France; 4 Vector Group Liverpool School of Tropical Medicine, Liverpool, United Kingdom; University of Crete, Greece

## Abstract

**Background:**

In the city of Yaoundé in Cameroon malaria is predominately transmitted by the M and S molecular forms of *Anopheles gambiae* and both are resistant to the pyrethroid insecticides and DDT. Mutations in the target site of these insecticides, present at a high frequency in malaria vectors in this city, contribute to this resistance profile. To identify additional resistance mechanisms, the expression profile of multiple DDT-resistant field populations of M and S molecular forms was compared to laboratory-susceptible populations.

**Methodology/Principal findings:**

The prevalence of DDT resistance was highest in the S form population originating from the cultivated site of Nkolondom (mortality after WHO bioassay = 4%). A high prevalence of DDT resistance was also found in two urban M form populations, Messa from a pristine unpolluted environment (DDT mortality = 54%), and Gare, where the breeding sites are heavily polluted with organic matter (DDT mortality = 38%). Microarray analysis showed that several transcripts coding for detoxification enzymes (P450s, GSTs and UDPGTs) and ABC transporters were upregulated in the three populations. Despite the presence of multiple detoxification genes over expressed in the DDT-resistant subset of these field populations, only three were commonly over expressed in resistant populations from all three environments. Two of these genes, *CYP6M2* and *GSTD1-6*, encode enzymes that have been previously shown to metabolize DDT.

**Conclusion/Significance:**

Analogous to target site resistance, genes involved in metabolic resistance to DDT are also shared between the M and S forms of *An gambiae*. Alternative explanations for this occurrence are explored.

## Introduction

Both insecticide treated nets (ITNs) and indoor residual spraying (IRS) are recommended by the World Health Organisation (WHO) [1], [2] for malaria control. Historically, organochlorine insecticides, in particular DDT, were the insecticide of choice for IRS across Africa [3], [4], [5]. Even today, although the majority of IRS in Africa uses pyrethroid insecticides, DDT is still in use in several countries [2].

In Cameroon, DDT was extensively used in the malaria eradication campaign in the 1950s through to the 1960s before being replaced by pyrethroids [3], [6], [7]. Since the introduction of pyrethroids in the early 1990s, IRS has been discontinued and replaced by the use of insecticide treated materials for malaria prevention across the country [3]. Both DDT and pyrethroid resistance are widespread in malaria vectors in Cameroon [8], [9], [10], [11]. Furthermore, with the intensification of urban agricultural practices in the late 1990s, the use of insecticides by farmers escalated. These insecticides frequently leech into surrounding water which may include mosquito breeding sites [10], [11]. As a consequence, malaria vectors from agricultural cultivated sites are often found to have higher levels of insecticide resistance than elsewhere [12], [13]. This was recently observed in two cities in Cameroon, where urban agriculture was associated with a higher prevalence of DDT resistance in malaria vectors [12].

The most well studied DDT resistance mechanism is target site resistance. Two alternative substitutions at codon 1014 in the sodium channel result in the replacement of leucine with either phenylalanine or serine [14], [15]. Both of these sodium channel mutations have been reported in Cameroon [10], [16], [17], [18] and more recently an additional mutation causing an asparagine to tyrosine substitution at codon 1575 was reported at a low frequency in the S form of *An. gambiae* from Yaoundé [19]. Both cytochrome P450 monooxygenases (P450s) and glutathione S-transferases (GSTs) have also been implicated in DDT resistance in *An. gambiae*. The Epsilon GST, GSTE2 and enzymes resulting from two alternative transcripts of the *GSTD1* gene are able to catalyse the dehydrochlorination of DDT to DDE [20], [21], and two cytochrome P450s, CYP6Z1 and CYP6M2, have been shown to metabolize DDT in vitro [22], [23]. Other putative DDT resistance mechanisms have been identified from microarray screens of *An. gambiae s.l.* populations but have not yet been functionally validated [24].

In Yaoundé, the predominant malaria vector is *An. gambiae s.s.* represented by it two molecular forms M and S. The two forms belong to the forest chromosomal form which is characterized by the absence of polymorphic inversions [25], [26]. Recent findings reported a segregation of the two forms distribution according to an ecological gradient correlated with the degree of urbanization [27]. DDT resistance is prevalent in both forms and is associated with a high frequency of kdr alleles [12], [18]. To determine whether additional mechanisms might be contributing to the resistance phenotype and to assess the similarity of these mechanisms between M and S forms, the transcription profile of DDT resistant mosquitoes, emerging from multiple breeding sites throughout the city of Yaoundé was examined.

## Materials and Methods

### Mosquito collections

Mosquito collections were conducted in three districts of the city of Yaoundé (3° 51′N 11° 30′E) the capital city of Cameroon. The city belongs to the Congo-Guinean phytogeographic domain characterized by a typical equatorial climate comprising two rainy seasons extending from March to June and from September to November with an annual rainfall of 1,700 mm. Samples were collected from multiple water bodies in three sites: Nkolondom, a district situated on the outskirts of the city characterized by vast lowland areas used for urban farming, Gare district, situated in the city centre and characterized by the high prevalence of polluted breeding sites and Messa district, situated within the city centre where the majority of the breeding sites are pristine, non polluted sites. Studies conducted in the city of Yaoundé revealed that, the M form is more prevalent in highly urbanized areas and less frequent in periurban or rural areas while in the other hand, the S form is largely predominant in rural and periurban areas and scarce in highly urbanized zones [27]. Further description concerning the properties of the *An. gambiae* breeding sites found in Yaoundé have been published previously [12]. The study was conducted under the ethical clearance N° 216/CNE/SE/09 delivered by the Cameroon National Ethics Committee Ref N° IORG0006538-IRB00007847-FWA00016054.

### Insecticide bioassays

Bioassays were conducted with F_0_ females obtained from field collected larvae. Mosquitoes were morphologically identified as members of the *An. gambiae* complex both at the larval and the adult stages using morphological identification keys [28]. Two to four days- old unfed *An. gambiae s.l.* were exposed to 4% DDT in WHO susceptibility test kits according to standardized procedures [29]. Mortality was recorded 24 hours post-exposure and mosquitoes were retained for a further 24 hours prior to storage in RNAlater. Tests with untreated papers were simultaneously run as controls. Mortality rate in tested samples was corrected using Abbot formula [30] when the mortality rate of control was between 5–20%. WHO criteria [29] were used to evaluate the resistance and susceptibility status of the tested mosquito population.

### Genotyping for *kdr* alleles

Genomic DNA used for molecular analysis was extracted from desiccated adult mosquitoes using the Livak method [31]. *An. gambiae* mosquitoes were identified to species and molecular form by PCR [32] protocol. The DDT resistant mosquitoes were screened for the presence of the *kdr* alleles (L1014F and L1014S) and the recently identified N1575Y sodium channel mutation using TaqMan assays [33] [19].

### Microarray experiments

Total RNA was extracted from batches of 10 mosquitoes using the PicoPure RNA isolation Kit (Arcturus). Three biological replicates were used for each population. Extracted RNA was treated with DNase (RNase free DNase set, Qiagen). RNA concentration and quality were assessed using nanodrop spectrophotometer (Nanodrop Technologies UK) and Bioanalyser (Agilent Technologies). 100 ng total RNA samples were amplified and labeled with Cy-3 or Cy-5 dye using the “Two color low input Quick Amp labeling kit”(Agilent technologies, Santa Clara, CA, USA) according to the manufacturer's instructions. Samples were purified (RNA purification kit, Qiagen) and cRNA labeling and yield were checked using a spectrophotometer (a NanoDrop Technologies) and Bioanalyzer (Agilent Technologies). Labeled cRNAs were hybridized to the Agilent 8×15 k ‘*Anopheles gambiae*’ array (AGAM_15K; full details provided at http://www.ebi.ac.uk/arrayexpress) (A-MEXP-2196) [33]. Hybridizations were conducted for 17 hours at 65°C at 10 rpm rotation and washed following the manufacturer's protocol (Agilent Technologies). Scanning of each microarray slide was performed with the Agilent G2565 Microarray Scanner System using the Agilent Feature Extraction Software (Agilent Technologies).

Comparison in all experiments consisted of three independent biological replicates and two dye swaps making a total of five hybridizations per comparison. Resistant samples from Messa and Gare were competitively hybridized with the Ngousso laboratory strain, all belonging to the *An. gambiae* M molecular form. S form Nkolondom samples were competitively hybridized to the S form Kisumu strain.

### Microarray data analysis

Microarray data were analysed using Genespring GX 11.1 software (Agilent Technologies).

Mean transcription expression ratios were submitted to a one sample student's t-test against zero with a Benjamin and Hochberg multiple testing corrections [34]. For each comparison transcripts showing both t-test p values<0.01 and a fold change ≥2-fold over or under transcribed were considered significantly and differentially transcribed compared to the susceptible strain. Lists of transcripts found significantly overtranscribed for the three comparisons were submitted to David 6.7 (http://david.abcc.ncifcrf.gov/) to detect functional categories significantly enriched.

### Microarray validation by qRT-PCR

Quantitative RT PCR was used to validate the microarray data following the protocol described in Bariami et al. [35]. Two micrograms of total RNA per biological replicate were reverse transcribed into cDNA using superscript III and oligo-dT20 primer (Invitrogen) according to the manufacturer's instructions. Quantitative PCRs were performed using a MX3005 Agilent system (Agilent). Each 25 µl reaction contained iQ SYBR Green supermix (Biorad), containing 0.3 µM of each primer and 5 µl of 1∶50 diluted cDNA. Melt curve analysis was used to verify the specificity of PCR products and serial dilutions of cDNA were used to generate standard curves for each gene. The fold change of selected transcripts, normalized to actin (AGAP000651_RA) and 40S ribosomal protein S7 (AGAP010592_RA), and relative to the susceptible strain (Ngousso or Kisumu), were calculated according to the 2^−ΔΔCT^ method incorporating PCR efficiency [36], [37]. The data have been deposited in Array express (accession number E-MTAB-1382).

## Results

### Species identification and kdr genotyping

Molecular species identification on a sample of 229 DDT survivors and control (not exposed to the insecticide) identified all specimens as *An. gambiae s.s.* The data was consistent with previous studies reporting the presence of only *An. gambiae* in the city of Yaoundé [25], [26], [38]. The M molecular form was predominant in both Messa (n = 36/36) and Gare (n = 70/79) while the S molecular form was the main form collected in Nkolondom (n = 106/114). The Ngousso and Kisumu laboratory colonies showed a mortality rate of 92 and 98% respectively after DDT exposure. Field populations showed DDT resistance with a mortality rate of 54.3% (n = 208) in Messa, 38% (n = 79) in Gare and 3.9% (n = 128) in Nkolondom (Table 1). The 1014F *kdr* allele was detected in all three sites, and the population from Nkolondom was fixed for this allele (Table 1). The N1575Y mutation was detected in the heterozygote state in only 3 S form specimens (all DDT resistant) after screening 134 specimens (M and S forms) in both survivors and control individuals.

**Table 1:** Molecular forms distribution, mortality after exposition to DDT 4% and Kdr allele frequency in *An. gambiae* populations from Yaoundé.

Collection sites	N	Molecular forms	% Mortality	Freq L1014F in survivors
Nkolondom	128	S	3.9%	100%
Messa	208	M	54.3%	29%
Gare	79	M	38%	78%
Ngousso	100	M	92%	
Kisumu	100	S	98%	

N, number of mosquitoes processed.

### Microarray data analysis

**Figure 1:**
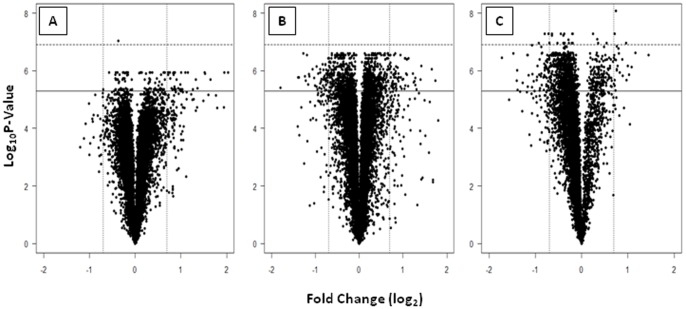
Volcano plots showing differential gene expression profile between DDT resistant and susceptible populations. Pairwise comparison between A: Ngousso vs Messa (M form, unpolluted site), B: Ngousso vs Gare (M form, polluted site) and C: Kisumu vs Nkolondom (S form, cultivated site). Vertical lines indicate 2 fold expression difference in either direction (−1>log2FC>1). The plain horizontal line indicates significance threshold (P<0.01).

**Figure 2:**
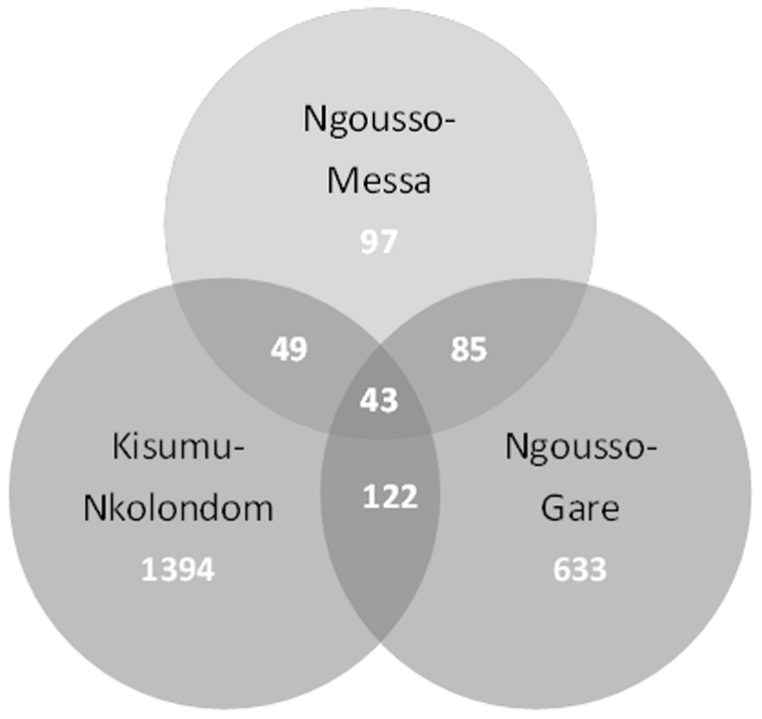
Genes differentially transcribed in each population (Genes showing a transcription ratio ≥2-fold in either direction comparatively to the susceptible strain and a corrected Pvalue<0.01). The number of transcripts is indicated for each area of the Venn diagram. Nkolondom (agricultural cultivated sites), Gare (polluted sites), Messa (unpolluted sites).

**Figure 3:**
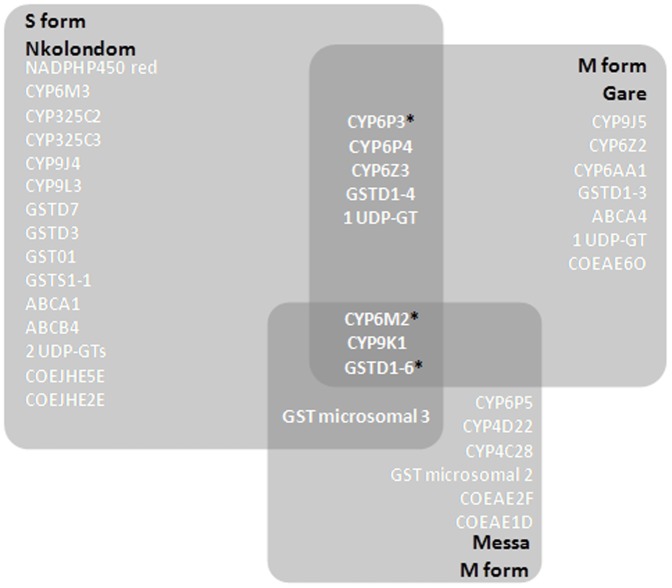
Venn diagram showing the insecticide resistance candidate genes over-transcribed in at least one condition. Enzymes proven to metabolize insecticides are marked with an asterisk. Size of the squares is correlated to the level of DDT resistance.

DDT resistant individuals from the three sites were competitively hybridized with either the S form Kisumu strain (Nkolondom) or the M form Ngousso strain (Gare and Messa). Global gene expression changes of genes differentially transcribed are shown in Figure 1 in the form of volcano plots. The data have been deposited in Array express (accession number E-MTAB-1382). Using the pre-determined cut off for significance and fold change, a higher number of differentially transcribed genes were identified in experiments using the S form (1608 transcripts in the Kisumu versus Nkolondom comparison (846 up regulated and 762 down regulated)) compared to the experiments using the M form (883 (409 up regulated and 473 down regulated) and 274 (194 up regulated and 80 down regulated) transcripts in the Gare vs Ngousso and Messa vs Ngousso comparisons respectively). This likely reflects the geographical origin of the susceptible strains with the Ngousso strain originating from Cameroon whereas the Kisumu strain is from Kenya. Several sets of genes including P450 monooxygenase (CYP), glutathione S transferase (GST), carboxylesterase (COE), glucosyl glucuronosyl transferase (UDPGT), cuticular proteins, extracellular transporters, energy metabolism proteins were recorded differentially transcribed. Forty six transcripts were up-regulated in DDT resistant populations from all three sites and 299 transcripts were found up-regulated in two of the three sites (Figure 2). Candidate detoxification genes found upregulated in both M and S forms included *CYP6M2, CYP9K1, CYP6P3, CYP6P4, CYP6Z3, GSTD1-6* and *GSTD1-4* (Table 2). A subset of seven transcripts was selected to validate the results of the microarray using qPCR and the results confirmed the up-regulation of the majority of transcripts. Apart from *CYP6M2* in Nkolondom samples which showed a significant difference in expression between qPCR and microarray, the fold changes were relatively closed between the two methods for the other validated genes (Table 3). Two genes, *CYP6M2* and *GSTD1-6*, encode enzymes that have previously been demonstrated to metabolize DDT and hence it is likely that the over expression of these genes contribute to the DDT resistant phenotype in both M and S form of *An. gambiae* in Yaoundé. Several additional detoxification genes such as *CYP6P3*, *CYP6Z3* are upregulated in the DDT resistant individuals from Gare (M form) and Nkolondom (S form) although a role for these in DDT metabolism has yet to be demonstrated (Figure 3).

**Table 2:** Transcript ID and putative functions of genes showing the highest over expression rate in comparisons between Messa (unpolluted) vs Ngousso (Ngus), Gare (polluted) vs Ngousso (Ngus) and Nkolondom ((Nkol) cultivated sites) vs Kisumu (Kis); FC: Fold Change.

		S form		M form			
		Kis-Nkol		Ngus-Gare		Ngus-Messa	
Transcript ID	Description	F C	Pvalue	F C	Pvalue	F C	Pvalue
AGAP008212-RA	CYP6M2	10.15	0.002	5.06	0.002	3.01	0.009
AGAP000818-RA	CYP9K1	3.96	0.005	3.67	0.003	4.13	0.009
AGAP004380-RA	GSTD1-6	2.52	0.007	2.01	0.004	3	0.003
AGAP002865-RA	CYP6P3	3.36	0.001	8.24	0.008		
AGAP002867-RA	CYP6P4	4.5	0.008	6.36	0.002		
AGAP007990-RA	glucosyl glucuronosyl transferases	3.24	0.001	8.66	0.004		
AGAP004164-RC	GSTD1-4	3.15	0.005	2.01	0.006		
AGAP008217-RA	CYP6Z3	3.11	0.006	3.86	0.001		
AGAP005837-RA	COEJHE5E	6.1	0.004				
AGAP006379-RA	ABCA1	4.67	0.002				
AGAP009946-RA	GSTMS3	4.61	0.001			3.9	0.009
AGAP008213-RA	CYP6M3	4.11	0.003				
AGAP002417-RA	CYP4AR1	3.93	0.004				
AGAP013121-RB	glucosyl glucuronosyl transferases	3.57	0.002				
AGAP006364-RA	ABCB4	3.34	0.002				
AGAP004163-RA	GSTD7	3.29	0.004				
AGAP005834-RA	COEJHE2E	3.27	0.009				
AGAP004382-RA	GSTD3	2.98	0.002				
AGAP012293-RA	CYP9L3	2.78	0.009				
AGAP000500-RA	NADPH P450 reductase	2.76	0.009				
AGAP007588-RA	glucosyl glucuronosyl transferases	2.23	0.008				
AGAP005749-RA	GSTO1	2.06	0.003				
AGAP008437-RA	ABCC8	2.01	0.003				
AGAP012296-RA	CYP9J5			7.99	0.003		
AGAP008218-RA	CYP6Z2			5.76	0.003		
AGAP011518-RA	ABCA4			5.42	0.009		
AGAP004164-RD	GSTD1-3			2.84	0.005		
AGAP005753-RA	glucosyl glucuronosyl transferases			2.7	0.002		
AGAP002863-RA	COEAE6O			2.63	0.003		
AGAP002862-RA	CYP6AA1			2.46	0.006		
AGAP002866-RA	CYP6P5					5.43	0.006
AGAP002419-RA	CYP4D22					3.87	0.009
AGAP010414-RA	CYP4C28					2.96	0.009
AGAP000163-RA	GSTMS2					2.13	0.003
AGAP006228-RA	COEAE2F					2.07	0.006
AGAP005756-RA	COEAE1D					2.7	0.003

**Table 3:** qPCR validation of microarray results.

	Nkolondom (Cultivated sites)			Gare (Polluted sites)			Messa (Unpolluted sites)		
	Microarray	qPCR		Microarray	qPCR		Microarray	qPCR	
Transcripts	F C	F C	95% CI	F C	F C	95% CI	F C	F C	95% CI
CYP6Z3	3.11	2.49*	2.3–2.7	3.86	3.6*	2.06–5.3	3.05 (NS)	7.54*	1.67–13.42
CYP6M2	10.15	78.12*	71.52–84.72	5.06	14.60*	4.5–24.7	3.01	9.25*	3.07–15.43
CYP6P3	3.36	13.73*	3.06–24.39	8.24	9.80*	4.8–14.76	5.37(NS)	5.6	0.85–12.07
CYP9K1	3.96	2.41	0.37–4.13	3.67	4.32*	3.13–5.52	4.13	2.47*	1.67–3.26
CYP6P4	4.5	5.69*	3.44–7.94	6.36	4.49*	1.82–7.17	5.4 (NS)	3.55	0.27–6.84
CYP6Z2	1.44 (NS)	4.65*	4.59–5.2	5.76	2.09*	2.05–2.26	3.43 (NS)	4.77	4.69–5.17
GSTD1-6	2.52	1.10	0.49–1.71	2.01	1.95*	1.64–2.25	3	4.15*	3.07–5.23

FC, fold change; NS, non significant;

* , Significant (confidence interval not overlapping with the susceptible); 95% CI, 95% confidence interval.

Detoxification genes were highly represented in the subset of genes up-regulated in DDT resistant populations. An enrichment analysis was performed to identify GO terms significantly enriched in the DDT resistant populations from the three sites, taking into consideration multiple testing corrections. GO terms designating Cytochrome P450s, glutathione S transferase, and superoxide dismutases were significantly enriched, with only P450s being enriched in the subset of genes up-regulated in DDT resistant individuals from all three sites (Table 4).

**Table 4:** GOTERM categories and functions of genes associated with the detoxification process found significantly enriched (terms showing a minimum count threshold of 2 and an Ease score (Pvalue<0.05); FE: Fold Enrichment).

Category	Go-terms functions	FE	Pvalue
**Over expressed in S form Nkolondom site**			
GOTERM_MF_FAT	GO:0004364 Glutathione transferase activity	16.24	1.1 E-03
INTERPRO	IPR001424 Superoxide dismutase, copper/zinc binding	12.67	2.0 E-02
GOTERM_MF_FAT	GO:0004784 Superoxide dismutase activity	12.18	2.2 E-02
GOTERM_MF_FAT	GO:0016721 Oxidoreductase activity acting on superoxide radicals as receptor	12.18	2.2 E-02
GOTERM_BP_FAT	GO:0006749 Glutathione metabolic process	11.66	2.4 E-02
GOTERM_BP_FAT	GO:0006801 Superoxide metabolic process	9.72	3.4 E-02
SP_PIR_KEYWORD	Postsynaptic cell membrane	5.05	5.7 E-03
SP_PIR_KEYWORD	Synapse	4.67	8.0 E-03
PIR_Super family	PIRSF000503 Glutathione S transferase	4.63	4.9 E-02
INTERPRO	IPR004045 Glutathione S transferase N terminal	4.22	1.2 E-02
GOTERM_CC_FAT	GO:0045211 Postsynaptic membrane	3.85	3.5 E-03
INTERPRO	IPR017933 Glutathione S transferase/chloride channel C-terminal	3.84	1.8 E-02
INTERPRO	IPR004046 Glutathione S transferase C-terminal	3.64	4.6 E-02
GOTERM_CC_FAT	GO:0044456 Synapse part	3.30	8.5 E-03
GOTERM_CC_FAT	GO:0045202 Synapse	2.95	1.6 E-02
INTERPRO	IPR017972 Cytochrome P450 conserved site	2.51	1.7 E-02
INTERPRO	IPR017973 Cytochrome P450 C-terminal region	2.48	1.9 E-02
INTERPRO	IPR001128 Cytochrome P450	2.37	1.7 E-02
SP_PIR_KEYWORDS	Monooxygenase	2.37	3.6 E-02
**Over expressed in Gare M form population**			
KEGG_Pathway	Aga00980: Metabolism of xenobiotic by cytochrome P450	17.17	4.0 E-03
KEGG_Pathway	Aga00982: Drug metabolism	14.71	1.5 E-02
INTERPRO	IPR004045: Glutathione S-transferase, N terminal	5.06	4.3 E-02
SP_PIR_KEYWORDS	Monooxygenase	4.43	9.1 E-04
INTERPRO	IPR017972: Cytochrome P450 conserved site	4.07	1.6 E-03
INTERPRO	IPR017973: Cytochrome P450 C-terminal region	4.02	1.7 E-03
INTERPRO	IPR002401: Cytochrome P450 E-class, group I	3.89	4.3 E-03
SP_PIR_KEYWORDS	Heme	3.51	4.0 E-03
INTERPRO	IPR002401: Cytochrome P450	3.49	4.1 E-03
GOTERM_MF_FAT	GO:0046906 Tetrapyrrole binding	2.72	6.7 E-03
GOTERM_MF_FAT	GO:0020037 Heme binding	2.72	6.7 E-03
**Over expressed in Messa M form population**			
INTERPRO	IPR002401: Cytochrome P450 E-class group I	5.03	1.7 E-02
INTERPRO	IPR017972: Cytochrome P450 conserved site	4.67	2.2 E-02
INTERPRO	IPR017973: Cytochrome P450 C-terminal region	4.62	2.2 E-02
INTERPRO	IPR001128: Cytochrome P450	4.00	3.5 E-02

## Discussion

Since the 2000s, a sharp increase in the prevalence of DDT resistance in malaria vectors have been reported across South Cameroon [10], [11]. This may be a result of cross resistance between DDT and pyrethroids, as an increase prevalence of pyrethroids resistance in mosquito populations has also been reported and target site mutations, known to confer resistance to both insecticide classes are common in this location. This study set out to determine whether additional mechanisms might be involved in DDT resistance in *Anopheles gambiae* M and S form populations in the city of Yaoundé.

DDT resistance was associated with an increase frequency of the 1014F *kdr* allele in samples from all study sites. The data was consistent with previous findings in South Cameroon [11], [16]. The 1575Y sodium channel mutation, which, when combined with 1014F, appears to result in higher levels of DDT resistance [19] was also detected at a low frequency in this study. The spread of this new mutation and its association with vectors resistance to insecticide needs to be carefully monitored.

Microarray analysis showed that several transcripts coding for detoxification enzymes (P450s, GSTs and UDPGTs) and ABC transporters were upregulated in the three populations. Several of these, including *CYP6M2*, *CYP9K1*, *CYP6P3*, *CYP6Z3*, *GSTD1-6* and *GSTD1-4*, were over transcribed in both *Anopheles gambiae* M and S molecular forms. The majority of these have been previously associated with resistance to pyrethroids and/or DDT [39], [40], [41], [42], [43]. Among cytochrome P450, *CYP6M2* and *CYP6P3*, found over-expressed in the three populations, have been functionally expressed and are capable of metabolizing both type I and type II pyrethroids [23], [41], [42], [43]. CYP6M2 was also found capable of metabolizing DDT and its predominant overexpression in the malpighian tubules supports a role in xenobiotic clearance [23]. Field studies reported a widespread distribution of this enzyme in pyrethroid resistant populations of *An. gambiae* in West Africa [39], [40]. *CYP6P3* has been found up-regulated in pyrethroid resistant populations of both *An. gambiae* and *An. arabiensis* [44] and its ortholog *CYP6P9* in *An. funestus* is also known to metabolize pyrethroids [41], [43]. A role for this enzyme in DDT metabolism has not yet been demonstrated. However it might contribute to the high prevalence of mosquito population resistance to both DDT and pyrethroids in the city of Yaoundé [12], [13]. Other *CYP6* candidate genes involved in DDT metabolism in M and S form populations include *CYP6Z3* found upregulated by both microarray and qPCR analysis. A member of the same subfamily *CYP6Z1* is able to metabolize DDT in vitro [22] however CYP6Z3 requires validation.

Certain glutathione transferases can cataylse the dehydrochlorination of DDT, and *GSTD1-6*, a splice variant of *GSTD1* [45] found over-expressed in all three populations, is known to possess this DDTase activity [46]. Additional genes from families with putative roles in insecticide resistance, such as UDPGTs, cuticular proteins, energy metabolism and transporters [47], [48], [49], [50], were found overexpressed in the DDT resistant populations from Cameroon. UDPGTs were over represented in both M and S molecular forms. Although their role in conferring resistance to insecticides is still under investigation, they have been frequently reported constitutively over expressed in insecticide resistant populations of *Drosophila, Anopheles* and *Aedes* [51], [52], [53]. Moreover, in mammals, they play an important role in the phase II detoxification processes [54] [55].

*An. gambiae* distribution modeling across Cameroon showed its two molecular forms to overlap to a large extent in the rainforest area where they occur in sympatry [38]. Genetic structure studies on populations from the city of Yaoundé reported limited gene flow between molecular forms [25]. Ecological and spatial analysis at the level of this city indicated the two forms to be markedly segregated along an urbanization gradient forming a bimodal cline with the M form more prevalent in densely urbanized setting and it densities decreasing from urban to rural areas whereas the S form was predominant in rural areas and less frequent in urban areas [27]. Despite restricted gene flow between molecular forms, several detoxification genes were over expressed in both M and S molecular forms. This may suggest an ancestral shared polymorphism that predisposes both forms to over expression of these genes. This is consistent with the recent divergence of molecular forms and the fact that differentiation between forms is still limited to a few genomic regions [56]. Alternatively the presence of common resistance mechanisms in both molecular forms could also highlight the influence of both concerted evolution and natural selection which, by homogenizing genes or loci not directly involved in reproductive isolation, may limit variability. Several lines of evidence support substantial contemporary gene flow between forms in areas of sympatry, particularly for genes under selection [57], [58]. The co-occurrence of the *kdr* alleles (1014F and 1014S) in the M form and the distribution of *Ace-1R* G119S mutation in both M and S molecular forms were reported to have occurred through introgression events in West Africa [59], [60], [61], [62].

Some differences in gene expression profiles were recorded between sites and could reflect the different genetic background between M and S forms or selection by different larval habitats. Samples originating from Nkolondom, situated in an area with intense agricultural cultivation, had the largest number of differentially expressed genes compared to its susceptible comparator. DDT resistance is at the highest level in this site and it is tempting to speculate that the transcription profile of this strain, is at least in part, related to the larval breeding site. In Cameroon, several insecticides including pyrethroids, carbamates, organochlorine and organophosphates, have been reported to be used in agricultural cultivated sites [11]. Most of the compounds used by farmers contaminate breeding habitats and are responsible for insecticide selection. A relationship between the practice of market gardening and increased vector resistance to insecticides has been demonstrated in several sites across Cameroon and in West Africa [11], [12], [63]. However, the use of an S form susceptible population from a different geographical region may be the primary explanation for this observation.

In addition to insecticides, several compounds including organic wastes, petroleum products or heavy metals were reported to contaminate anopheline breeding habitats and are believed to induce cross resistance to insecticides [53], [64], [65]. As such it is expected that mosquitoes emerging from polluted environment possess a high variety of detoxification mechanisms able to metabolize a large range of substances. Consistent with these observations a greater number of genes were differentially expressed in the site of Gare where breeding sites are for the majority organically polluted sites, than in the relatively pristine Messa site. In this case, both comparisons were between the same molecular form and using the same, susceptible population originating from the same city. Experimental analysis using *Aedes aegypti* reported selection by organic xenobiotics to induce overproduction of transcripts encoding cuticular proteins while selection by heavy metals such as copper induced overexpression of detoxification enzymes [66]. The fact that several sets of detoxification genes were found upregulated in DDT resistant mosquitoes emerging from polluted environments perhaps suggests selection by different types of pollutants in the natural environment.

## Conclusion

The data derived from this study suggest similar resistance profile between *An. gambiae* M and S molecular forms in the city of Yaoundé and stress the need to understand resistant mechanisms associated with vector resistance in order to improve vector control strategies in the field. Some detoxification enzymes, including *An gambiae CYP6M2* and *CYP6P3*, show a broad substrate specificity and can confer cross resistance to multiple insecticide classes. The identification of this, and other enzymes with proven roles in insecticide resistance, up-regulated in multiple populations of *An gambiae* from the capital city of Cameroon is concerning and requires appropriate measures such as the switch to other insecticide families or where necessary the association of larval control to mitigate the impact that pyrethroid resistance might have on the efficiency of impregnated nets.
